# High‐Conductivity Electrolytes Screened Using Fragment‐ and Composition‐Aware Deep Learning

**DOI:** 10.1002/advs.202521575

**Published:** 2026-01-04

**Authors:** Xiangwen Wang, Muyang Chen, Gengyi Bao, Yan Lai, Jinghe Cao, Xinhua Liu, Rui Tan

**Affiliations:** ^1^ Department of Physics and Astronomy University of Manchester Manchester UK; ^2^ Department of Chemical Engineering Swansea University Swansea UK; ^3^ School of Transportation Science and Engineering Beihang University Beijing China; ^4^ School of Chemistry Tiangong University Tianjin China

**Keywords:** battery electrolyte, data‐driven design, graph neural networks, ionic conductivity, machine learning

## Abstract

Rising energy generation from renewables (e.g., wind, solar power) will drive global demand for >1.0 TWh of long‐duration energy storage by 2030 to stabilise grids and balance supply. Rechargeable batteries are central to this transition, with their performance critically governed by the properties of active materials and supporting electrolytes. However, designing electrolyte formulations remains a major challenge, as their performance arises from complex, non‐additive interactions among lithium salts and organic solvents, requiring elegant molecular design and selection. Conventional trial‐and‐error strategies still dominate electrolyte design, but they are slow and resource‐intensive. Recent machine learning approaches have improved electrolyte screening, yet many rely on coarse molecular representations that neglect fragment‐level chemistry and explicit ratios, limiting interpretability and their utility for guiding experiments. Here we introduce a deep learning framework that integrates intermolecular attributions across solvents with intramolecular attributions from functional units. The framework builds a hierarchical representation, decomposing formulations into molecules and their functional units, while integrating ratios, physicochemical descriptors, and salt identity to generate mixture‐invariant embeddings for accurate and interpretable conductivity prediction. Applied to benchmark datasets of lithium battery electrolytes, the framework achieves high accuracy in predicting ionic conductivity and enables large‐scale virtual screening. Crucially, it provides chemically interpretable insights: fragment‐level attentions align with functional units; composition‐aware attention reveals the impact of mixing ratios; and counterfactual perturbations confirm causal roles of key motifs. This framework paves the way for data‐driven, interpretable electrolyte design and can be generalized to broader formulation challenges in materials science.

## Graphical abstract

We present a new deep learning framework that hierarchically links molecular and functional unit attributions to predict electrolyte conductivity. By integrating molecular composition, ratios, and physicochemical descriptors, it achieves accurate, interpretable predictions and large‐scale virtual screening, offering chemically meaningful insights for data‐driven electrolyte and formulation design across materials science.

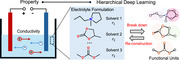

## 1 Introduction

Battery electrolyte is a unique component, as it interfaces with every other part and must simultaneously satisfy multiple constraints: rapid ion transport, electronic insulation, and stability against electrodes [1, 2, 3, 4, 5]. Historically, the design of electrolytes that are highly conductive, non‐flammable, and stable across wide electrochemical windows is a central challenge in modern battery chemistry, i.e., a challenge that extends beyond lithium‐ion to sodium‐ion, flow, and other battery systems [6]. Ionic conductivity is a central performance metric in electrolytes, governing ion transport, charge‐discharge kinetics, and degradation processes such as metal plating, stripping, and interfacial impedance [7, 8]. Limited ionic conductivity has been shown to constrain battery performance under demanding conditions, including low‐temperature operation, high‐rate cycling, and ‘anode‐free’ configurations [9, 10, 11]. At the molecular level, ionic conductivity is strongly influenced by electrolyte composition, salt concentration, and solvation structure [12]. Comprehensively exploring this multidimensional formulation space requires varying multiple components and concentrations, leading to a vast number of possible combinations.

Given this complexity, electrolyte formulation has been investigated using a variety of experimental and computational strategies [13, 14, 15]. Electrolyte discovery has historically relied on trial‐and‐error experimentation, which is resource‐intensive and time‐consuming [16, 17]. Deep learning is widely used across battery research [18, 19, 20], and its extension to the more specific task of electrolyte design is both natural and powerful [21, 22]. Earlier strategies, including design‐of‐experiment methods, surrogate models, or descriptor‐based regression, achieved partial success but relied on hand‐crafted features and treated mixtures as simple concatenations, limiting their ability to capture non‐additive interactions [23, 24, 25]. Recent progress in molecular representation learning, particularly graph neural networks (GNNs) [26] and chemical foundation models [27], now allows models to operate directly on molecular structures and learn task‐specific features without manual descriptors. Despite this progress, applications to formulation modeling, whether through set‐based architectures or foundation models, have largely remained at the mixture or molecule level. These approaches overlook the fine‐grained structural basis of ion transport, making it difficult to attribute performance to specific substructures or composition ratios.

Here, we introduce a hierarchical attention framework that simultaneously captures intramolecular features reflecting the chemistry and geometry of individual molecules, and intermolecular interactions governing non‐additive mixture properties. Each molecular substrate is first transformed into atom‐level embeddings by a GNN encoder, which are then pooled into fragment embeddings through subgraph masking to represent functional units (FUs). Composition ratios, physicochemical descriptors, and salt identity are subsequently integrated via composition‐aware attention to reconstruct a formulation embedding for predicting ionic conductivity. To evaluate model performance and generalization, we collected two independent datasets of electrolyte formulations with corresponding ionic conductivity measurements. The framework was benchmarked against baseline models on each dataset. Beyond predictive performance, the hierarchical attention architecture provides chemically interpretable insights into electrolyte behavior. Intermolecular attention reveals which solvents and salts dominate conductivity, while intramolecular attention highlights the FUs that facilitate ion transport. This interpretability connects model outputs to chemical intuition, enabling data‐driven understanding and rational formulation design. Theoretically, this protocol can be extended beyond predicting liquid electrolyte to designing highly conductive solid‐state electrolytes, an even more critical area in need of such technology.

## 2 Result and Discussion

### 2.1 Data Collection

**FIGURE 1:**
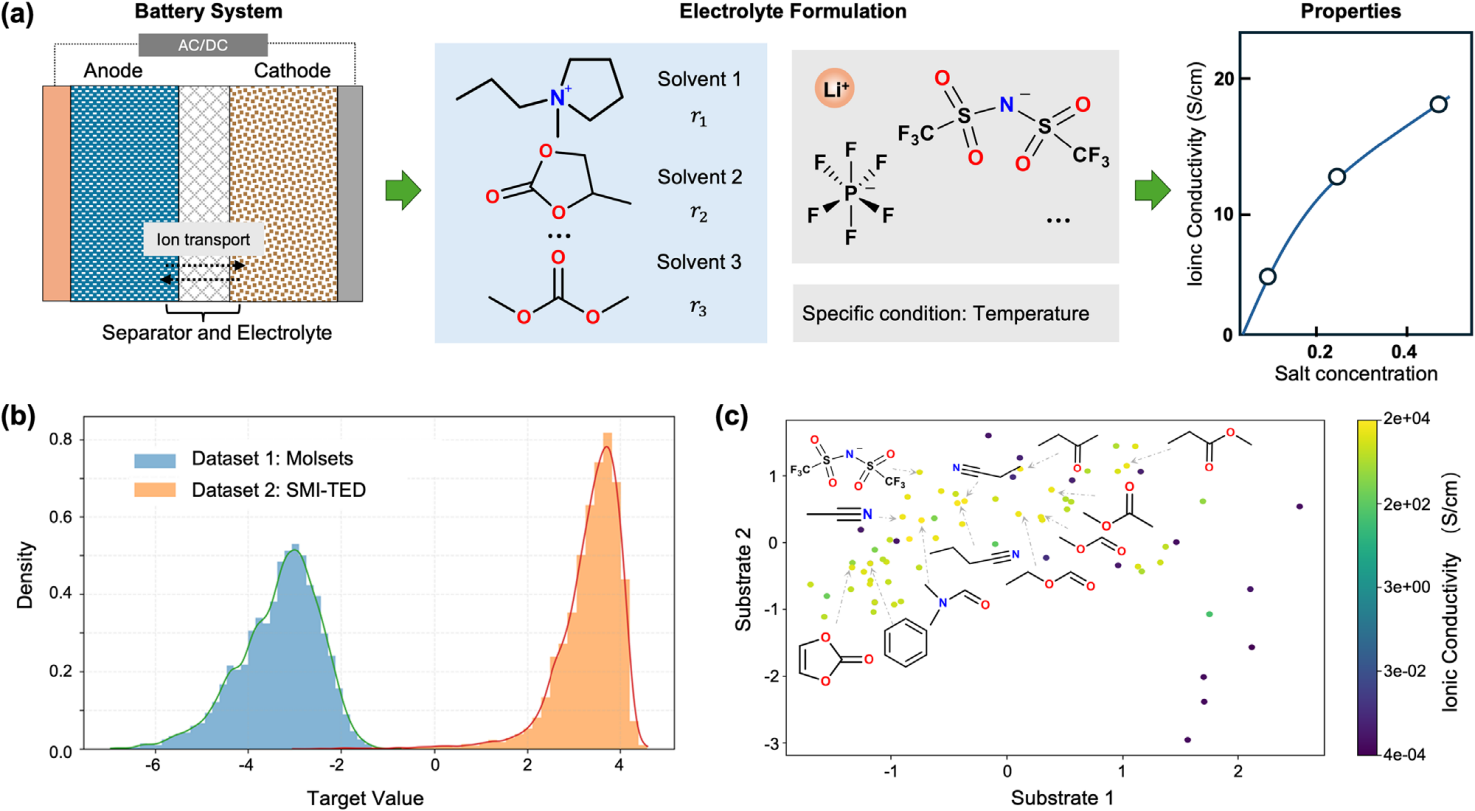
Analysis of electrolyte formulation datasets. (a) Schematic of the task: predicting the macroscopic property (conductivity) from an electrolyte formulation that combines several molecular components with molar ratios and a salt, under specified conditions (e.g., temperature). (b) Distribution of the target value landscape for the MolSets (blue) and SMI‐TED (orange) datasets. (c) Visualization of the substrate structure space, where each point corresponds to a molecule and is colored by ionic conductivity (log scale). Representative chemical structures with high conductivity are shown to highlight structural diversity.

Figure 1 summarizes the prediction task and compares the two electrolyte datasets, MolSets [26] and SMI‐TED [28]. We formulate the task as predicting ionic conductivity directly from electrolyte formulations composed of a lithium salt and one or more solvents with specified molar ratios under given conditions (e.g., temperature and salt level), shown in Figure 1a. This setting reflects the non‐linear composition‐property relationships that govern electrolyte performance. To investigate model behavior across different chemical spaces, we selected two complementary datasets that differ in composition coverage, measurement range, and formulation complexity. MolSets reports ionic conductivities for a wide range of solvent–salt pairs. Although the dataset is typically visualized in terms of weight fractions, creating the appearance of compositional variation, the underlying experimental protocol fixes every binary solvent mixture at a 1:1 molar ratio, as specified in the original study. Because molecular weights differ substantially between solvents, converting this fixed molar ratio into weight fractions yields different apparent values across samples. Importantly, this variation is an artifact of representation rather than a true compositional degree of freedom: all MolSets formulations share exactly the same molar ratio. Since our model operates on true molar compositions rather than weight‐fraction displays, MolSets does not provide meaningful ratio diversity for learning composition–property relationships. Instead, its value lies in a different aspect: MolSets offers a chemically diverse but stoichiometrically fixed collection of formulations, allowing us to test whether the model can generalize across different molecular identities even when the composition is held constant. In this sense, MolSets serves as a benchmark for evaluating robustness under fixed‐ratio conditions, complementing datasets like SMI‐TED that contain genuine composition variability. In contrast, the SMI‐TED dataset is both larger and compositionally expressive. Each formulation contains explicit solvent ratios, spanning one to five components with truly variable mole fractions, which we convert to molar percentages (mol%) using reported densities and molecular weights. SMI‐TED explores diverse recombination and continuously varying compositions within a narrower family of solvents, effectively spanning a high‐dimensional mixture‐design space. Consequently, the two datasets exhibit fundamentally different target value landscapes (Figure 1b).

Figure 1c visualizes the molecular structure space constructed from the both dataset, where each point corresponds to a solvent molecule colored by its associated target value. The embedding reveals that chemically similar solvents cluster together, yet conductivity values vary substantially within local regions, underscoring the highly non‐linear structure–property relationships. Notably, high‐conductivity molecules appear in multiple distinct regions of the space, indicating that favorable transport properties emerge from diverse chemical motifs.

### 2.2 Model Construction and Data Representation

**FIGURE 2:**
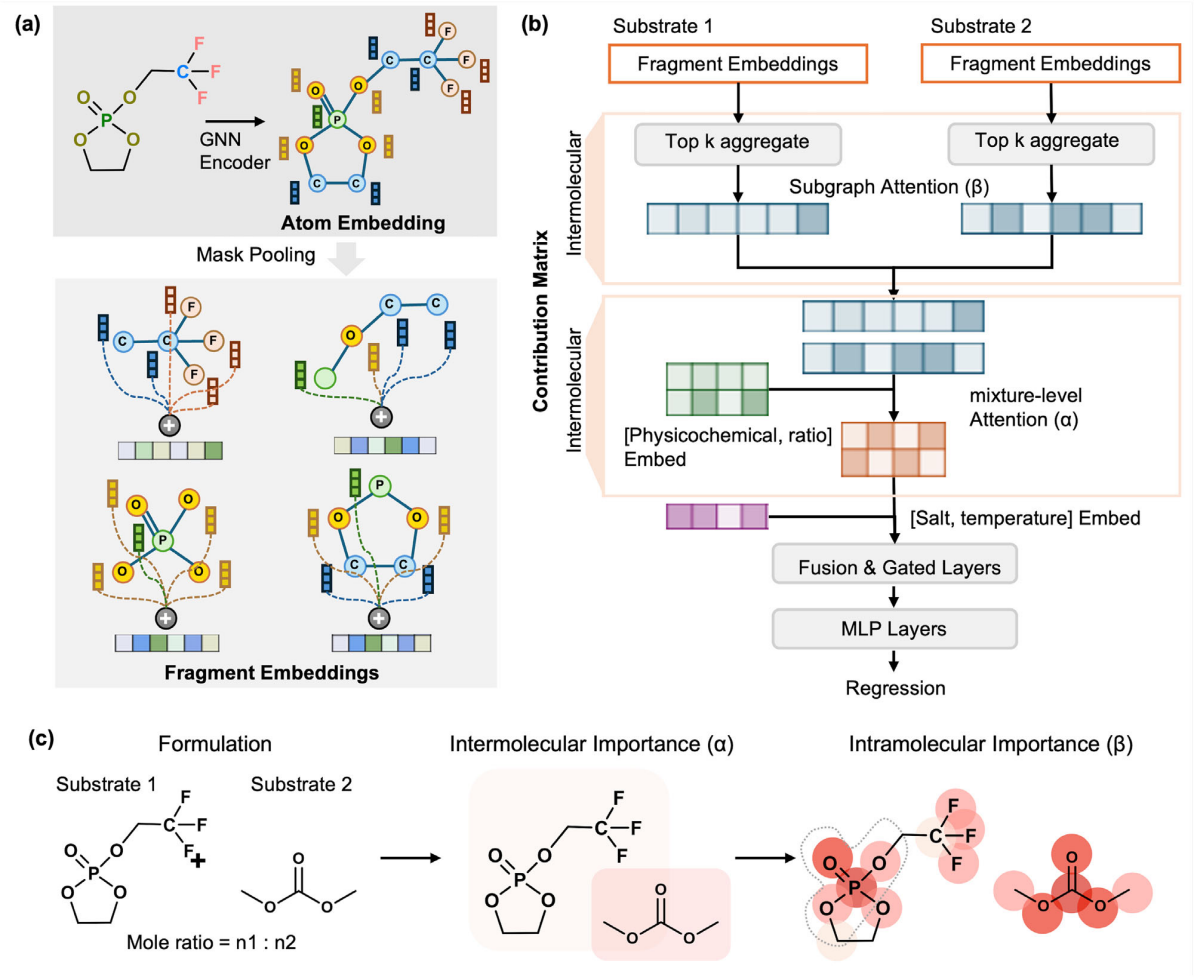
Overview of the proposed hierarchical structure‐informed framework for interpretable electrolyte formulation modeling. (a) Structure‐informed molecular representation, illustrating how each molecule is decomposed into chemically meaningful fragments from atomic structures. (b) Architecture of the hierarchical graph–attention model for electrolyte formulations, consisting of an intramolecular and an intermolecular attention module. (c) Hierarchical α–β attribution illustrating component‐ and fragment‐level importance in an electrolyte formulation.

Figure 2 provides an overview of our hierarchical framework, which combines structure‐informed data representations with a graph–attention model to predict ionic conductivity from electrolyte formulations.

To represent the electrolyte formulation, each substrate is first expressed as a molecular graph where atoms and bonds define the structural backbone and encoded with a GNN module to produce atom‐level embeddings. To move beyond atom‐wise features, we introduce subgraph masks that pool atom embeddings into chemically meaningful fragments reflecting FUs, enabling the model to capture localized environments most relevant to ion transport. This design grounds the representation in interpretable chemical units rather than treating each molecule as an indivisible entity, shown in Figure 2a.

Figure 2b shows the structure of the proposed hierarchical graph–attention model. The fragment embeddings serve as inputs. Within each molecule, attention is applied over fragment embeddings to assign intramolecular importance scores to structural motifs, after which they are aggregated into component‐level embeddings. These component embeddings are then passed to a mixture‐level bilinear attention module that assigns intermolecular importance score while integrating two additional information channels: mixture ratios, which ensure that relative proportions of components influence the representation, and physicochemical descriptors (e.g., polarity, dielectric constant, molecular weight), which provide global priors complementing structural features. The resulting formulation‐level embedding is further conditioned on salt identity and temperature, thereby capturing both compositional effects and cross‐component interactions that govern ionic conductivity. This hierarchical design enables the model to reason across molecular scales, linking atomic and functional‐unit chemistry with macroscopic mixture behavior, and providing physically interpretable insights into electrolyte performance.

During inference, the hierarchical attention mechanism yields two levels of attribution that collectively explain a formulation's predicted conductivity. For a given electrolyte formulation, the mixture‐level bilinear attention produces intermolecular importance scores (α), quantifying the contribution of each molecular component to the overall property. Within each component, the fragment‐level attention provides intramolecular importance scores (β), highlighting the FUs and structural motifs most responsible for ion transport. Together, they form a component–fragment contribution matrix that disentangles the relative importance of solvents and their functional motifs, as shown in (Figure 2c).

This hierarchical design offers two advantages. First, subgraph pooling makes the learned representation inherently interpretable, allowing predictions to be traced back to chemically meaningful fragments. Second, integrating mixture ratios and physicochemical descriptors equips the model to handle heterogeneous formulations and extrapolate across composition regimes. As shown in later experiments, these innovations are essential for robust performance across both constrained (MolSets) and diverse (SMI‐TED) datasets.

### 2.3 Deep Learning Model Performance

**FIGURE 3:**
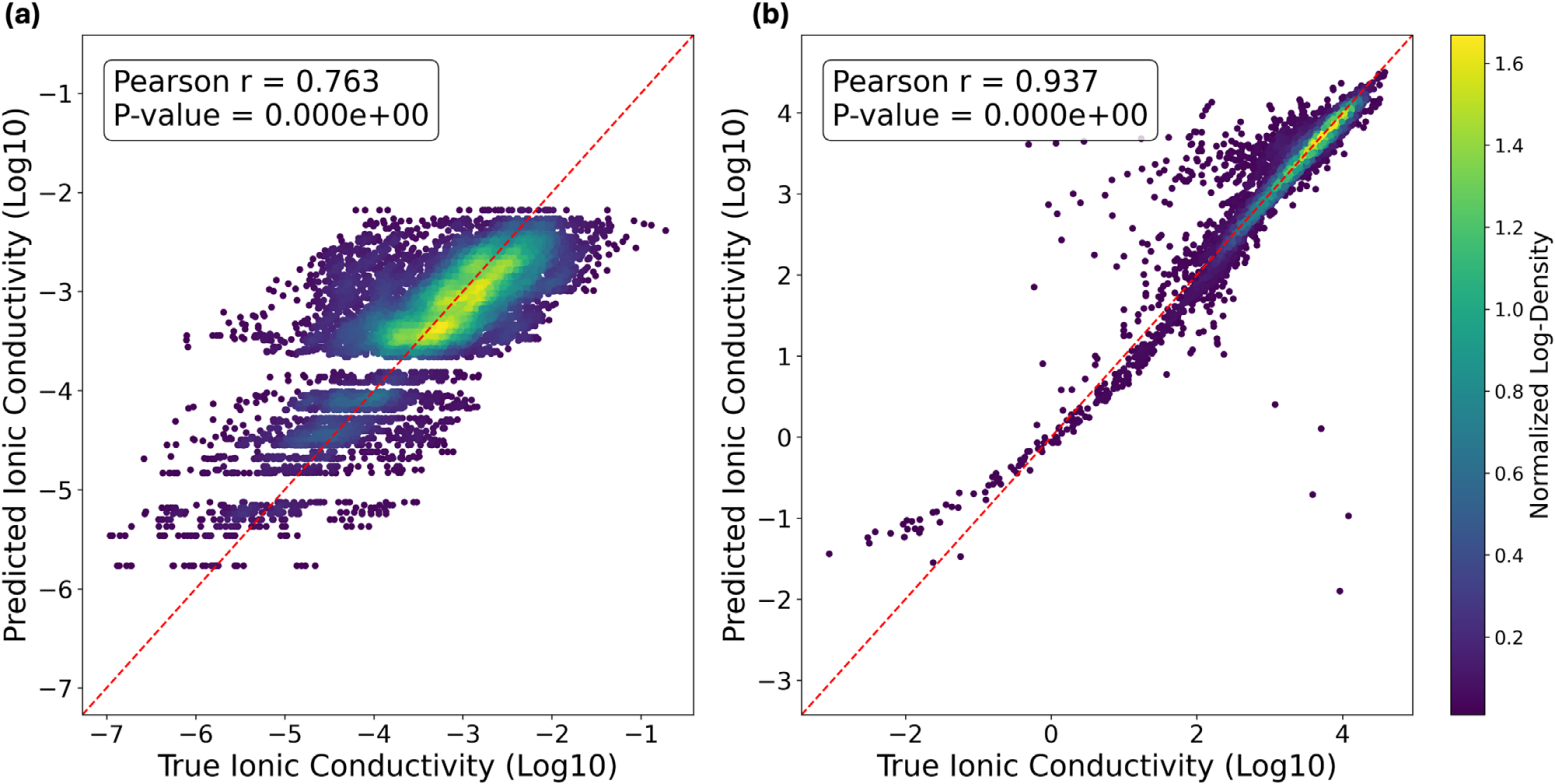
Regression plots showing the target values (horizontal axis) and predicted values (vertical axis) of logarithmic conductivity (a) Molsets dataset. (b) SMI‐TED dataset.

We evaluated the predictive performance of our framework on the two prototypical electrolyte datasets: MolSets and SMI‐TED, shown in Figure 3. On MolSets (Figure 3a), the model achieves a Pearson correlation of 0.763 between predicted and experimental ionic conductivities, reflecting reasonable agreement despite the dataset's limited size and constrained conditions. On the more diverse and larger SMI‐TED dataset (Figure 3b), performance improves substantially, yielding a Pearson correlation of 0.937 and capturing a broad dynamic range of conductivities. To support the learned formulation trends, we further performed atomistic MD simulations on representative MF–EC mixtures (details in Section S2), which confirmed the same composition‐dependent behavior observed in our model predictions.

To benchmark our model, we compared it against both existing formulation models and a series of progressively simplified architectures. Two recently proposed methods serve as external references: MolSets, which represents mixtures as permutation‐invariant sets of molecular graphs and aggregates them through a Deep Sets architecture with attention, and SMI‐TED, a chemical foundation model fine‐tuned on a large collection of electrolyte conductivity data, where formulations are expressed as concatenated SMILES strings (Simplified Molecular Input Line Entry System), augmented with composition and temperature. Both methods capture mixture‐level properties but do not explicitly resolve the contribution of structural fragments within molecules. In addition, four internal baselines were introduced to strip away different levels of structural information. PhysChemLinear predicts conductivity using only physicochemical descriptors aggregated by molar ratio, serving as a non‐graph feature baseline. SimpleMean adds atomic and fingerprint embeddings but averages them without graph convolutions. GCNMean incorporates molecular graph structure through stacked Graph Convolutional Network (GCN) layers, followed by ratio‐weighted averaging. DeepSets adapts the Deep Sets paradigm by embedding each component with its physicochemical features and ratio, transforming them with a shared network, and summing the outputs. Together, these baselines allow us to disentangle the incremental effects of structural encoding, ratio‐awareness, and set‐based aggregation relative to our full hierarchical model.

Table 1 summarizes the benchmarking results across MolSets and SMI‐TED. Our model consistently outperforms simplified internal baselines (PhysChemLinear, SimpleMean, GCNMean, and DeepSets) and also exceeds the original MolSets architecture, demonstrating the benefits of subgraph‐level molecular encoding and the hierarchical α–β attribution mechanism. When evaluated on SMI‐TED, our single, lightweight model achieves comparable accuracy to the SMI‐TED framework, despite the fact that SMI‐TED is built on a large chemical foundation model pre‐trained on 91 million molecules. In contrast, our method does not rely on massive pretraining; instead, it derives predictive power directly from chemically meaningful FUs and interpretable subgraph structures. Although designed for interpretability rather than maximal accuracy, our model still achieves competitive performance while providing mechanistic insights at the formulation, molecular, and functional‐unit levels. Overall, it offers a concise and chemically interpretable alternative to foundation‐model approaches.

**TABLE 1:** Benchmark studies of the performance of machine learning models in regression tasks, using the Molsets dataset, SMI‐TED dataset.

Dataset	Molset dataset		SMI‐TED dataset	
Models	*RMSE*	*R* ^2^	*RMSE*	*R* ^2^
DeepSets	0.585	0.526	0.696	0.134
GCNMean	0.682	0.357	0.692	0.143
PhysChemLinear	0.776	0.166	0.872	0.075
SimpleMean	0.707	0.309	0.694	0.139
Molsets	0.588	0.649	0.651	0.146
SMI‐TED	—	—	0.108	0.977
**Ourmodel**	**0.560**	**0.566**	**0.248**	**0.920**

* SMI‐TED is based on a SMILES Transformer Encoder–Decoder model, pre‐trained on 91 million molecules and subsequently fine‐tuned for the prediction task. Since the original implementation did not release code, only the results reported in the paper are available.

### 2.4 Formulation and Structural Correlations with Conductivity

**FIGURE 4:**
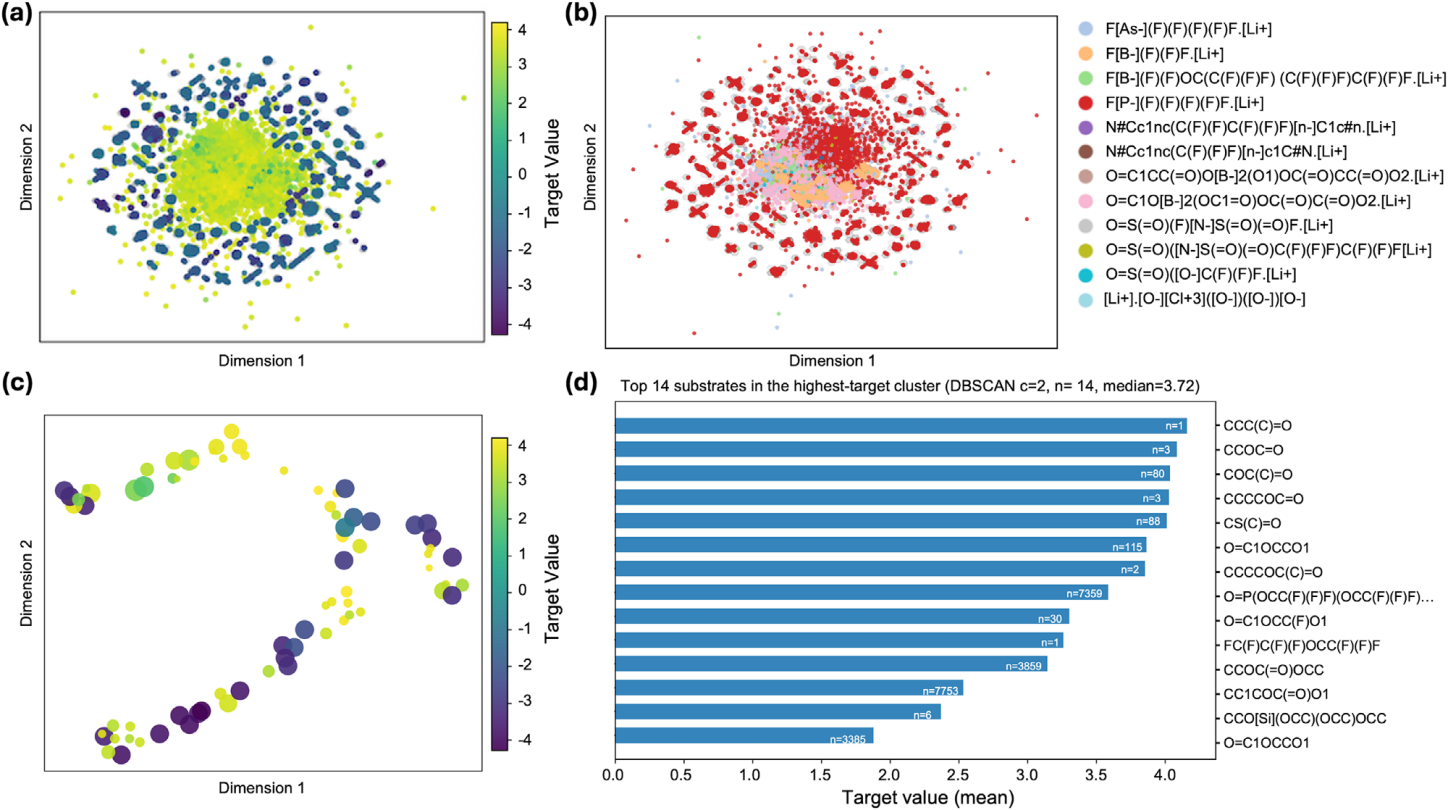
Formulation‐ and substrate‐level embeddings visualized with UMAP. (a) Formulation‐level mixture embeddings colored by experimental conductivity. (b) Formulation‐level UMAP space colored by salt identity, highlighting how different salts populate distinct regions of the embedding. (c) Unique substrate molecules extracted from formulations, with points colored by the associated target value and sized by their number of appearances in the dataset. (d) Top substrates enriched in the high‐target cluster, ranked by their mean target value.

In this section, we first analyze the formulation‐level embedding space learned by the model. Specifically, we extract the mixture embeddings from the output of the mixture‐level attention module and project them into two dimensions using principal component analysis (PCA) for preprocessing, followed by Uniform Manifold Approximation and Projection (UMAP) [29, 30] to visualize clustering patterns and chemical relationships among electrolyte formulations. In Figure 4a, the formulation‐level embedding space is colored by experimentally measured conductivity. The target values, shown in the range of [−4, 4], correspond to the z‐score standardized targets. A clear gradient is observed, where high‐conductivity formulations cluster in specific regions, while low‐conductivity ones are distributed toward the periphery. This indicates that the learned formulation embeddings capture global composition–property relationships that correlate with experimental performance. It is worth noting that the overall projection appears approximately circular and radially divergent. This shape does not correspond to the physical structure of the formulations, but rather reflects a typical artifact of UMAP visualization that UMAP tends to spread high‐dimensional embeddings evenly in low‐dimensional space, preserving local neighborhoods while globally balancing the layout, which often results in disk‐like or radiating patterns. Thus, the meaningful information lies in the relative clustering of points and the color gradient, rather than in the outer contour itself. In Figure 4b, the same embedding space is colored by the salt identity. Distinct salts occupy different regions of the space, with some salts enriched in the high‐conductivity zone while others cluster in the low‐conductivity area. This demonstrates that the embedding space not only encodes overall formulation–property relationships, but also reflects the critical role of salts in determining system performance. Specifically, Lithium hexafluorophosphate (LiPF_6_, red dots) dominates the distribution of data points, consistent with its widespread use as a lithium salt [31, 32, 33] and indicating the applicability and reliability of our deep learning framework. Interestingly, as shown in Figure 4a,b, lithium bis(oxalato)borate (LiBOB, pink dots) and lithium tetrafluoroborate (LiBF_4_, orange) cluster within high‐conductivity domains, suggesting that these salts can enhance the conductive performance of liquid electrolytes. This observation is consistent with previous reports, where the incorporation of LiBOB was shown to improve both ion transport and electrochemical performance [34, 35]. In contrast, lithium bis(fluorosulfonyl)imide (LiFSI, grey) and lithium (fluorosulfonyl)(perfluoropropanesulfonyl)imide (LiFTFSI, yellow) appear less frequently in the dataset, but still cluster in the high‐conductivity domain, consistent with their chemical nature: bulky, charge‐delocalized anions that weakly coordinate with Li^+^, promoting ion dissociation and mobility while suppressing ion pairing [36, 37].

Next, we performed a molecular‐level analysis, moving from the formulation space down to the individual substrates used within each mixture, shown in Figure 4c. All substrates appearing in the dataset were extracted and represented by their molecular embeddings, obtained from the GNN encoder followed by subgraph pooling. Embeddings corresponding to the same SMILES string were aggregated by averaging, yielding a unique representation for each distinct substrate. These unique substrate embeddings were standardized and projected into two dimensions using UMAP. In the visualization, color encodes the mean target value across all formulations in which a substrate appears, while point size reflects the number of occurrences. This allows us to identify both which substrates are more frequently associated with high or low target values, and which substrates are more common in the dataset. Substrates with a higher target mean value cluster in adjacent regions, which indicates that the learned molecular embeddings capture the link between structural differences at the substrate level and their macroscopic property contributions. Larger points correspond to more frequently used substrates, which also have greater influence on the overall organization of the embedding space. To further identify key contributors, we applied DBSCAN clustering [38] to the UMAP space of unique substrates. Among the resulting clusters, we selected the one with the highest median target mean value, which we interpret as a “high‐target enriched region.” Within this cluster, substrates were ranked, and the top‐ranked entries were displayed as a horizontal bar chart (Figure 4d). As predicted, solvents such as methylethylketone (CCC(C)═O), ethyl formate (CCOC═O), methyl acetate (COC(C)═O), n‐butyl formate (CCCCOC═O), and methyl thioformate (CSC═O) can enhance Li^+^ conductivity because their carbonyl or thioester groups strongly solvate Li^+^, while their low viscosity and favorable dielectric properties promote ion dissociation and transport [3]. High ionic conductivity and low viscosity alone do not ensure robust interphase formation or long‐term electrode compatibility [39, 40, 41, 42]. In contrast, typical solvents such as ethylene carbonate (EC, O═C1OCCO1), diethyl carbonate (DEC, CCOC(═O)OCC), fluoroethylene carbonate (FEC, O═C1OCC(F)O1), and more recently widely used heavily fluorinated solvents such as HFE‐type ethers (FC(F)C(F)(F)OCC(F)(F)F) and FTEP (O═P(OCC(F)(F)F)(OCC(F)(F)F)OCC(F)(F)F) generally contribute to lower ionic conductivity, as their high viscosity, strong Li^+^ coordination, and in the case of fluorinated species, reduced dielectric constant hinder ion dissociation and slow down Li^+^ transport. These results further confirm the reliability of our deep learning framework and reasonably suggest that electrolyte formulations combining predicted salts (e.g., borate‐based salts) with solvents containing carbonyl or thioester groups can deliver higher ionic conductivity.

Beyond the molecular level, our framework enables interpretation at an even finer granularity, the level of FUs. This hierarchical design makes it possible to trace how each chemically meaningful fragment contributes to the overall conductivity prediction, thus revealing how local chemical environments within molecules influence ion‐transport behavior. For each formulation, the trained model was run once while computing gradient–times–input scores on each molecule's subgraph embeddings. FUs are defined by SMARTS patterns with compact labels (e.g., ─tBu, ─OMe, ─NO_2_). All FUs that appear at least once in the dataset are automatically included based on these SMARTS definitions. Within each molecule, we collected the matched heavy (non‐hydrogen) atoms corresponding to a given FU and identified all fragments whose center atom fall within this set. The signed fragment‐level attention scores (β) are then averaged to obtain a single within‐molecule effect for that FU, where positive values indicate that the FU contributes to higher predicted conductivity, and negative values indicate a suppressing effect. This fragment‐level effect is subsequently weighted by the model's mixture attention for the corresponding molecule (α), yielding one contribution value per formulation–molecule–FU triplet. Finally, all contribution values across the dataset were globally standardized to facilitate comparison and visualization of FU‐level effects.

**FIGURE 5:**
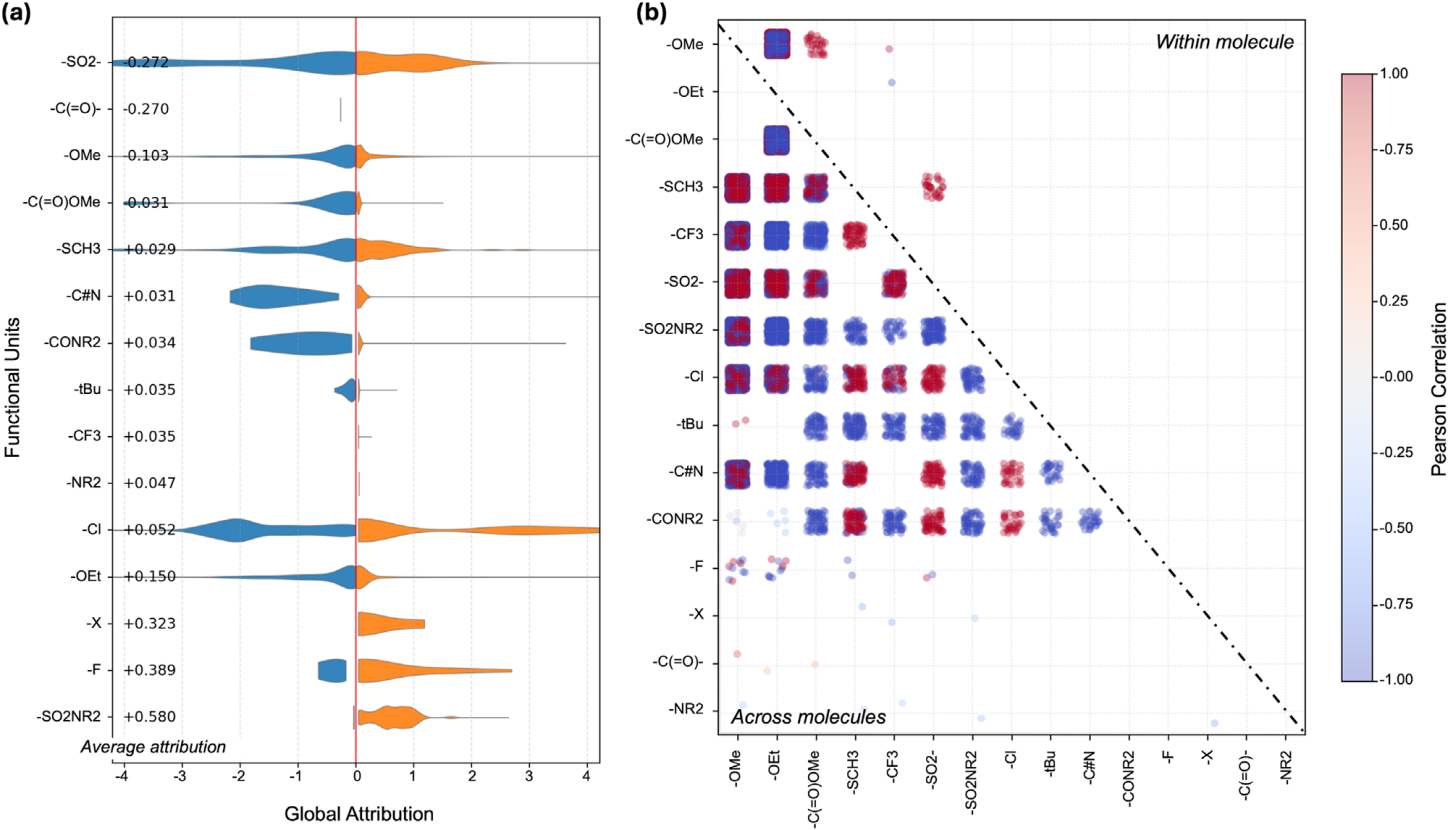
Functional–unit attribution and pairwise interactions. (a) Split‐violin distributions of global attribution for unique FUs. For each FU, densities to the left of zero (blue) indicate negative standardized contributions and densities to the right (orange) indicate positive contributions. The numeric mean value is shown on the left. (b) Pairwise correlation across all formulations. Colors encode the correlation (blue = negative, white ≈ 0, red = positive). The upper triangle shows correlations computed from molecules in which the two FUs co‐occur. The lower triangle shows correlations computed across different molecules in the same formulation.

In Figure 5a, we interpret the split violins as standardized, dataset–level effects on the predicted ionic conductivity. Distributions that extend far from zero correspond to FUs that strongly modulate the model output, whereas narrow shapes tightly centered near zero indicate near–neutral influence. When both blue (negative) and orange (positive) lobes are substantial for the same FU, the effect is highly context dependent, flipping sign across mixtures as component identity, mixture ratios, and substrate physicochemical descriptors vary. In contrast, a consistently one‐sided violin suggests a more uniform tendency across the dataset. Applying this lens to our results, sulfone‐like (─SO_2_─) and carbonyl (─C(═O)─) motifs show clearly left‐shifted distributions with negative means (around −0.27), indicating that they are more often associated with reduced predicted ionic conductivity, while still exhibiting occasional positive tails in specific formulations. This result is in strong agreement with the top substrates listed in Figure 4d, where carbonyl groups are present in several solvents that generally contribute less to conductivity, with notable exceptions such as ethyl formate and methyl acetate, which enhance conductivity. Methoxy (─OMe) and ester carbonyl methoxy (─C(═O)OMe) display mildly negative tendencies. By contrast, several FUs skew positive, including thioether methyl (─SCH_3_), cyano (─C≡N), dialkyl amide (─CONR_2_), tert‐butyl (─tBu), trifluoromethyl (─CF_3_), dialkyl amino (─NR_2_), chloro (─Cl), ethoxy (─OEt), generic halogen (─X), fluoro (─F), and sulfonamide (─SO_2_NR_2_). Among these, ─F, ─X, and ─SO_2_NR_2_ exhibit particularly long right‐hand (orange) tails, reflecting a strong positive association with increased predicted conductivity in a substantial subset of mixtures. Overall, the figure provides a chemically interpretable overview that positively skewed FUs tend to appear in higher‐conductivity formulations, negatively skewed FUs in lower‐conductivity ones, while symmetric or tightly centered shapes signal weak or formulation‐specific effects. Importantly, these are standardized, model‐based attributions derived from the product of mixture‐level attention α and per‐molecule FU subgraph attribution β; they represent relative, context‐dependent contributions captured by the model rather than absolute physicochemical constants.

In mixtures, FUs rarely act in isolation; their effects can reinforce or counteract each other depending on whether they reside on the same molecule or on different components. Figure 5b visualizes these pairwise interactions by plotting Pearson correlations of standardized FU attributions for every FU pair. Two regimes are shown in a single panel. The upper triangle (“Within molecule”) computes correlations using only molecules that contain both motifs, representing co‐occurring substructures on the same scaffold. The lower triangle (“Across molecules”) considers two motifs appear on different components within the same formulation, reflecting co‐variation across components within a mixture. Each dot represents one formulation colored by the correlation trend (blue = negative, white = near zero, red = positive), while local dot density reflects how frequently the pair appears. In the across‐molecule regime (lower triangle), most cells are pale and centered near zero, indicating weak or formulation‐specific trade‐offs when motifs reside on different molecules. Nevertheless, dense red or blue patches mark pairs that repeatedly move together or in opposition across many formulations. In contrast, within molecules (upper triangle), clearer chemistry trends emerge: oxygenated motifs often co‐vary positively, e.g., −OMe with ‐OEt or ‐C(═O)OMe shows compact red clusters—consistent with tending to rise or fall together on the same scaffold. Halogenated groups (‐Cl, −CF_3_, ‐F) also skew positive with one another, while thioether methyl (−SCH_3_) and sulfone‐like (−SO_2_ −) pairs frequently show blue clusters against oxygenated partners, suggesting antagonistic contributions when these motifs co‐occur. Overall, dense red (blue) clusters highlight FU pairs that consistently correlate positively (negatively) in the model's assigned importance, whereas empty or whitish cells indicate little systematic interaction. Thus, our model elucidates interactions among FUs within and across molecules, indicating that the design of high‐conductivity electrolyte formulations requires avoiding antagonistic pairs, such as combining oxygenated groups with sulfur‐based partners.

## 3 Conclusion

We introduce a structure‐informed deep learning framework that predicts electrolyte conductivity directly from formulation composition and molecular structure. The hierarchical architecture captures intramolecular functional‐motif effects reflecting the chemistry and geometry of individual molecules, and intermolecular interactions that govern non‐additive mixture behavior, achieving state‐of‐the‐art predictive accuracy across benchmark datasets without reliance on massive pretraining. Beyond numerical performance, the framework provides interpretable attributions at three hierarchical levels: formulation, revealing composition‐dependent behavior; molecular, quantifying individual component contributions; and functional‐unit, resolving the local chemical motifs that modulate ionic conductivity. These multi‐level attributions provide direct chemical insights. Analyses of formulation and molecular embedding spaces highlight the dominant role of salts such as LiPF_6_ and reveal that borate‐based salts (LiBOB, LiBF_4_) and weakly coordinating imides (LiFSI, LiFTFSI) are consistently associated with high‐conductivity domains. At the solvent level, carbonyl and thioester groups promote Li^+^ transport through strong solvation and low viscosity, whereas carbonate and heavily fluorinated species suppress conductivity due to high viscosity and strong ion pairing. Functional‐unit attribution further uncovers synergistic and antagonistic motif interactions that dictate conductivity. Closely aligned with the research direction of this field [43], our framework establishes a general paradigm for modeling complex formulations and can be readily extended to the rational design of advanced electrolytes and other multicomponent material systems where microscopic interactions govern macroscopic function. Beyond demonstrating predictive capability, it thus offers broadly applicable guidance for next‐generation battery chemistries. Of great note, ionic conductivity represents only one facet of electrolyte optimization. While essential, it is insufficient on its own. Practical electrolyte design requires the simultaneous evaluation of electrochemical stability, interfacial compatibility, volatility, viscosity and safety. Incorporating these additional objectives will guide the continued refinement of our framework and underpin future advances in predictive electrolyte design.

## 4 Experimental Section

### 4.1 Preprocessing Pipeline

Molecular structures were converted from SMILES into RDKit [44] graphs with explicit hydrogens. Each atom was assigned a categorical index based on its element, with aromatic atoms treated as a separate token. A global dictionary dynamically maps observed atom types to integer indices; atoms exceeding the vocabulary are mapped to a default token. This yields an atom feature array $a_{i}\in {\left\{0,\text{…},A-1\right\}}^{N_{i}}$ for molecule *i*. Bond types (single, double, aromatic, etc.) are similarly indexed, giving an adjacency matrix $A_{i}\in {\left\{0,\text{…},B-1\right\}}^{N_{i}\times N_{i}}$. To capture local structural environments, atom‐centered fingerprints are constructed using a Weisfeiler–Lehman neighborhood expansion up to radius *r*. At each step, labels are updated by combining the atom label with those of bonded neighbors, then mapped to integer identifiers through a subgraph dictionary. This yields fingerprint indices $f_{i}\in {\left\{0,\text{…},F-1\right\}}^{N_{i}}$. Fragment‐level pooling is enabled by binary subgraph masks. For each heavy atom, a *k*‐hop neighborhood is extracted (with optional bonded hydrogens), producing a mask vector of length *N*_*i*_. Collecting all heavy‐atom neighborhoods yields a mask matrix $S_{i}\in {\left\{0,1\right\}}^{S_{i}\times N_{i}}$, where *S*_*i*_ is the number of heavy atoms. An electrolyte formulation is represented as an unordered set of molecular entries:

(1) $$M={\left\{\left(G_{i},f_{i},S_{i},p_{i},w_{i}\right)\right\}}_{i=1}^{M}$$

where *G*_*i*_ = (**a**_*i*_, **A**_*i*_) encodes atom and bond features, **f**_*i*_ are fingerprints, **S**_*i*_ are subgraph masks, $p_{i}\in R^{P}$ are physicochemical descriptors, and *w*_*i*_ is the mixture ratio of component *i*. The target property (ionic conductivity) is permutation‐invariant with respect to component ordering.

### 4.2 Model Structure

The model predicts mixture‐level properties from sets of molecular graphs with associated descriptors and ratios. Atoms and fingerprints are embedded into a hidden dimension *H*, while bond types are encoded separately. A stack of *L* GINEConv blocks (message passing, residual connection, layer normalization, SiLU, dropout) produces node embeddings $H_{i}\in R^{N_{i}\times H}$.

Given subgraph masks, masked multi‐head attention aggregates node features into subgraph embeddings. For subgraph *s*,

(2) $${\tilde{s}}_{i,s}=W_{O}\cdot \mathrm{Attn}\left(Q,K,V;{\text{mask}}_{s}\right)\in R^{H}$$

with query, key, and value projections defined as **Q** = **H**_*i*, ref_ **W**_*Q*_, **K** = **H**_*i*_ **W**_*K*_, **V** = **H**_*i*_ **W**_*V*_. A scalar score is then computed by

(3) $$u_{i,s}=\frac{{\tilde{s}}_{i,s}^{⊤}q_{\mathrm{sg}}}{\sqrt{H}}$$

We select the top‐*k* subgraphs $K_{i}$ and form a molecular embedding:

(4) $$m_{i}=\sum_{s\in K_{i}}\alpha_{i,s}^{\left(\mathrm{top}k\right)}\;{\tilde{s}}_{i,s},\;\alpha_{i,s}^{\left(\mathrm{top}k\right)}={\mathrm{softmax}}_{s\in K_{i}}\left(u_{i,s}\right)$$

For interpretability, we also expose intramolecular importance over all subgraphs:

(5) $$\beta_{i,s}={\mathrm{softmax}}_{s}\left(u_{i,s}\right)$$

indicating which fragments in molecule *i* are most influential.

At the mixture level, molecular embeddings **m**_*i*_ are fused with physicochemical descriptors **p**_*i*_ and normalized ratios ${\bar{w}}_{i}$. Each component builds a key:

(6) $$k_{i}=\mathrm{MLP}\left([p_{i}\;∥\;{\bar{w}}_{i}]\right)\in R^{H}$$

Component scores are computed as

(7) $$s_{i}=m_{i}^{⊤}W_{b}k_{i}\cdot \tau +\lambda_{\mathrm{prior}}\log\left(\max\left({\bar{w}}_{i},\varepsilon \right)\right)$$

followed by a softmax to obtain intermolecular importance:

(8) $$\alpha_{i}={\mathrm{softmax}}_{i}\left(s_{i}\right),\;\sum_{i=1}^{M}\alpha_{i}=1$$

The structural embedding is then

(9) $$z_{\mathrm{struct}}=\sum_{i=1}^{M}\alpha_{i}\;m_{i}$$

blended with a ratio‐weighted shortcut

(10) $$\tilde{z}=W_{\mathrm{simp}}\sum_{i=1}^{M}{\bar{w}}_{i}\;m_{i}$$

through a learnable gate:

(11) $$z=\left(1-\gamma \right)\;z_{\mathrm{struct}}+\gamma \;\tilde{z}$$

The mixture embedding **z** is passed through an MLP head to predict conductivity:

(12) $$\hat{y}=f_{\theta }\left(z\right)$$

The training objective combines mean squared error with an entropy regularizer on α:

(13) $$L=\frac{1}{B}\sum_{b=1}^{B}{\left({\hat{y}}^{\left(b\right)}-y^{\left(b\right)}\right)}^{2}-\lambda_{\mathrm{ent}}\sum_{b=1}^{B}\sum_{i=1}^{M}\alpha_{i}^{\left(b\right)}\log\alpha_{i}^{\left(b\right)}$$

### 4.3 Evaluation and Training Details

Model performance is assessed using 5‐fold cross‐validation. The dataset is randomly partitioned into five equal folds; in each run, one fold is held out for testing while the remaining four are used for training (with one fold optionally set aside for validation if early stopping is used). We report mean squared error (MSE), mean absolute error (MAE), and coefficient of determination (*R* ^2^) on the held‐out test fold, together with Pearson and Spearman correlation coefficients. Final results are reported as the mean and standard deviation across the five folds.

The deep learning model is implemented in Python 3.9, utilizing PyTorch 2.6.0 [45] for neural network construction, scikit‐learn 1.6.1 [46] for data preprocessing. Optimization is performed using Adam [47] with an initial learning rate of 10^−3^, weight decay of 10^−4^, and gradient clipping with maximum norm 2.0. The learning rate is decayed by a fixed multiplicative factor at user‐defined intervals. Batch size, hidden dimension *H*, number of GNN layers *L*, number of attention heads, and top‐*k* subgraphs are set from configuration files. Dropout with probability 0.1 is applied to all hidden layers. For each fold, the best checkpoint is selected based on validation *R* ^2^, and we report the averaged performance across the five folds to ensure robustness.

All models were trained either on a single NVIDIA Tesla P40 GPU (24 GB) using CUDA 12.4 or on a local CPU machine when applicable. A 40‐epoch training schedule was used for all experiments, and we empirically validated that 40 epochs are sufficient for convergence. The complete hyperparameter settings and training schedule are provided in the Section S1.

## Conflicts of Interest

The authors declare no conflicts of interest.

## Supporting information




**Supporting File**: advs73583‐sup‐0001‐SuppMat.pdf.

## Data Availability

All datasets and code are available at https://github.com/Xiangwen‐Wang/FragForm.
