# Supplementary material for: High‐Conductivity Electrolytes Screened Using Fragment‐ and Composition‐Aware Deep Learning
Source: Adv Sci (Weinh). 2026 Jan 4;13(14):e21575. doi: 10.1002/advs.202521575 (PMC12970172; doi:10.1002/advs.202521575)
Supplement: Supplementary file 1 — Supporting File: advs73583‐sup‐0001‐SuppMat.pdf. [file ADVS-13-e21575-s001.pdf]

Supplementary Information for

High-Conductivity Electrolytes Screened using Fragment- and Composition-Aware Deep Learning

Xiangwen Wang[a], Muyang Chen[b], Gengyi Bao[c], Yan Lai[d], Jinghe Cao[d], Xinhua Liu[c], Rui Tan[b,*]

[a]Department of Physics and Astronomy, University of Manchester, Oxford Road, Manchester, M13 9PL, United Kingdom

[b]Department of Chemical Engineering, Swansea University, Swansea, SA1 8EN, United Kingdom

[c]School of Transportation Science and Engineering, Beihang University, Beijing, 100191, China

[d]School of Chemistry, Tiangong University, Tianjin, 300387, China

*Corresponding author(s). E-mail(s): rui.tan@swansea.ac.uk

A Hyperparameter optimization

Table 1 summarizes the key hyperparameters explored for each model component—including the GNN, attention mechanism, and training strategy, together with their candidate values and functional descriptions. Each setting was trained for 40 epochs, and the corresponding training and validation performance curves are shown in Figure 3, evaluated on a representative subset of the data. These comparisons provide empirical support for the chosen configuration, highlighting its stability and consistent convergence across model variants.

Table 1 Summary of hyperparameter settings.

Module	Parameter	Options	Description
**GNN**	Hidden feature dimension	[64, **128**, 256]	Size of latent representation for node/edge embeddings
**GNN**	Number of layers	[3, **4**, 5]	Depth of stacked GINEConv layers
**Attention**	Number of heads	[2, **4**, 8]	Multi-head subgraph attention
**Attention**	Top-k subgraphs	[3, **4**, 5, all]	Number of most informative subgraphs aggregated per molecule
**Regularization**	Dropout rate	[0.05, **0.1**, 0.2]	prevents overfitting
**Training**	Learning rate	[**1e-3**, 5e-4, 2e-3]	Learning rate for optimization
**Training**	Batch size	[8, **16**, 32]	Size of each training batch

Bolded values indicate the selected configuration used in the final model, chosen based on validation performance on a representative data subset.

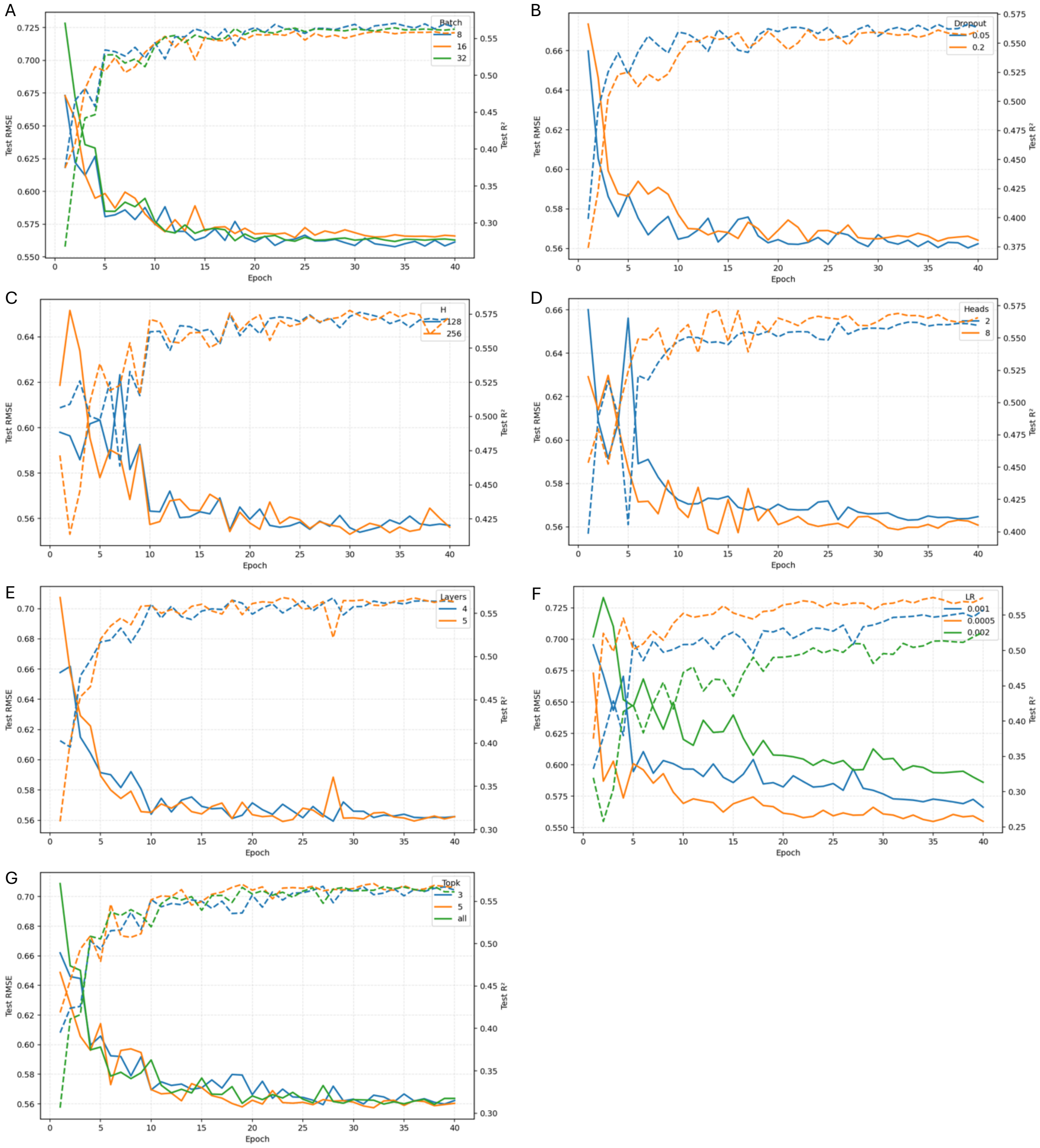

Figure 1 Validation performance under different hyperparameter configurations. Test RMSE (left panels) and R^2 score (right panels) for different settings of key hyperparameters: A. batch size, B. dropout value, C. GNN hidden feature dimension, D. number of pooling heads, E. GNN layer configuration, F. learning rate, and G. number of top subgraphs for aggregation. Results are evaluated on the MolSets dataset.

B Case study and Molecular Dynamics Validation

To examine whether the model captures physically meaningful formulation–structure relationships, we conducted atomistic MD simulations on an MF–EC binary solvent system. This system was selected because the model predicts a monotonic increase in ionic conductivity with increasing MF fraction:

Table 2 Prediction results of MF–EC binary solvent system.

**MF:EC (mol ratio)**	**Predicted $\log_{10}$(IC)**
**1:3**	2.54
**1:1**	2.67
**3:1**	1.80

B.1 Simulation Details

Molecular dynamics (MD) simulations were performed by the Gromacs 2024 program. Restrained electrostatic potential (RESP) charges computed by Multiwfn[S1,S2] and General Amber force fields (GAFF) parameters were adopted for all solvent molecules and ions. The GAFF-based topology files used for simulation processes were generated by the Sobtop program. [S2] Electrolytes were modeled in an initial cubic box with 5 nm length. The velocity-rescale thermostat method was used for temperature controlling. First, an energy minimization step was performed on the boxes to adjust the positions of molecules and ions and reduce the potential energy of the system. Then, the electrolyte boxes were pre-equilibrated in 100 ps NVT and 100 ps NPT ensembles, respectively. Finally, the electrolyte boxes were fully equilibrated under isolated conditions for 1 ns. The last 0.1 ns trajectories were sampled for radial distribution function (RDF) and coordination structure counting analyses. During the simulation processes, VMD was used for visualization.

B.2 Mechanistic Validation

Figure S2 displays RDFs and first-shell coordination numbers obtained from the MD trajectories. A clear compositional trend is observed: Li–O(MF) coordination increases steadily with increasing MF content; Li–O(EC) coordination decreases correspondingly; Li–anion coordination remains near zero, confirming the absence of contact-ion pairs or aggregates. This competitive solvation behavior directly supports the monotonic trend predicted by the model. MF is a lower-viscosity, more weakly coordinating solvent than EC; its increased presence in the first solvation shell produces a more weakly bound and dynamically exchanging Li^+ environment, consistent with higher local ion mobility. The directional trend agrees with prior experimental observations [S3], where MF-rich mixtures exhibit reduced viscosity,

weaker Li^+–solvent binding, and improved transport performance. Notably, the MD simulations focus on microscopic solvation structures rather than macroscopic conductivity, whose magnitudes depend on measurement conventions and long-timescale ion transport inaccessible to atomistic simulations.

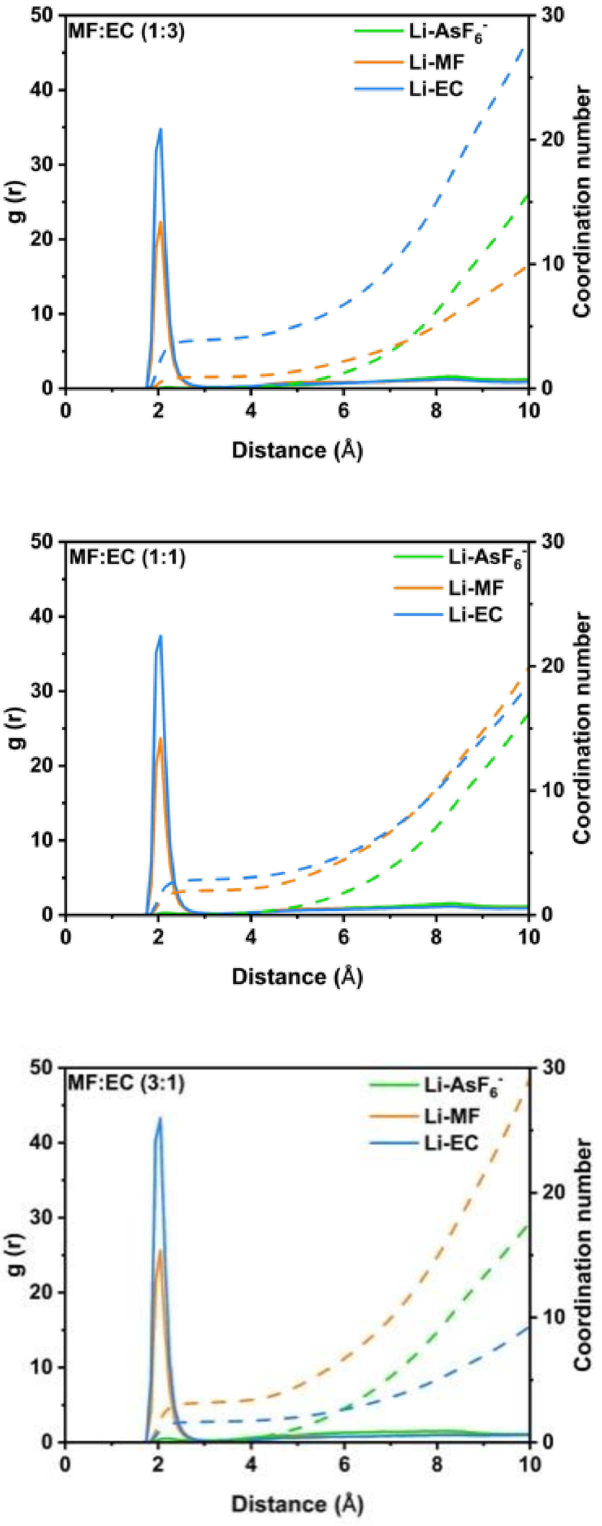

Figure 2 RDF and coordinate results of three formulations from MD simulation.

Reference:

[S1] Lu, Tian, and Fei-Wu CHEN. "Comparison of computational methods for atomic charges." Acta Physico-Chimica Sinica 28.1 (2012): 1-18.

[S2] Lu, Tian, and Feiwu Chen. "Multiwfn: A multifunctional wavefunction analyzer." Journal of computational chemistry 33.5 (2012): 580-592.

[S3] Ein-Eli, Y., et al. "Li-ion battery electrolyte formulated for low-temperature applications." Journal of the Electrochemical Society 144.3 (1997): 823.
